# A CAP–ALT–HbA1c Model for Biopsy-Confirmed Moderate-to-Severe Hepatic Steatosis in Bariatric Surgery Candidates: Development and Internal Validation

**DOI:** 10.3390/jcm15197493

**Published:** 2026-09-26

**Authors:** Jingli Ding, Mengjie Hu, He Wang, Cheng Gong, Zixuan Mei, Wanshun Chun, Dawei Deng, Zixuan Ma, Xiang Zhang, Qiu Zhao, Jianliang Zhou, Zhen Li

**Affiliations:** 1 Department of Gastroenterology, Zhongnan Hospital of Wuhan University, Wuhan 430071, China; dingjingli0216@163.com; 2 Department of Cardiovascular Surgery, Zhongnan Hospital of Wuhan University, Wuhan 430071, China; mengjiehu@whu.edu.cn (M.H.);; 3 Department of Hepatobiliary and Pancreatic Surgery, Zhongnan Hospital of Wuhan University, Wuhan 430071, China; wanghe@whu.edu.cn (H.W.);; 4 Bariatric and Metabolic Diseases Surgery Center, Zhongnan Hospital of Wuhan University, Wuhan 430071, China

**Keywords:** hepatic steatosis, bariatric surgery, controlled attenuation parameter, liver biopsy, HbA1c, risk stratification

## Abstract

**Background/Objectives:** The controlled attenuation parameter (CAP) is widely used for non-invasive assessment of hepatic steatosis, but its discrimination for moderate-to-severe steatosis may be limited in patients with obesity. We developed and internally validated a model combining CAP, alanine aminotransferase (ALT), and glycated haemoglobin (HbA1c) to identify biopsy-confirmed S ≥ 2 steatosis in bariatric surgery candidates. **Methods:** This retrospective single-centre study included 320 bariatric surgery candidates with preoperative CAP and laboratory data and intraoperative wedge liver biopsy as the reference standard. Candidate predictors were evaluated using univariable logistic regression, least absolute shrinkage and selection operator (LASSO) screening, and multivariable logistic regression. Model performance was assessed using receiver operating characteristic analysis, paired DeLong testing, bootstrap internal validation, calibration, Brier score, and decision curve analysis. **Results:** S ≥ 2 steatosis was present in 182 patients (56.9%). The combined model showed higher discrimination than CAP alone (area under the curve [AUC] 0.768, 95% CI 0.717–0.819 vs. 0.671, 95% CI 0.612–0.731), with an AUC difference of 0.096 (95% CI 0.049–0.144; paired DeLong *p* < 0.001). At the internally derived Youden threshold, sensitivity was 53.8% and specificity was 86.2%. The combined model also showed a lower Brier score than CAP alone (0.1941 vs. 0.2234). Bootstrap internal validation yielded an optimism-corrected AUC of 0.760 and a mean absolute calibration error of 0.017. Decision curve analysis suggested modest incremental net benefit over CAP alone. **Conclusions:** The CAP–ALT–HbA1c model showed higher discrimination and lower overall prediction error than CAP alone. It may complement CAP as an adjunctive tool for preoperative risk stratification, but external validation and assessment of clinically appropriate decision thresholds are required before routine clinical implementation.

## 1. Introduction

Metabolic dysfunction-associated steatotic liver disease (MASLD), formerly termed nonalcoholic fatty liver disease, is closely associated with cardiometabolic risk factors, including obesity, dysglycaemia, hypertension, and dyslipidaemia [1,2]. Bariatric surgery candidates represent a population with a high prevalence of hepatic steatosis [3]. Although body mass index (BMI) is widely used to characterize obesity severity, it does not directly quantify liver-specific fat accumulation [4]. Accurate assessment of liver fat burden may therefore provide clinically relevant information beyond measures of body size alone and help identify patients who warrant further liver and metabolic evaluation before surgery.

Hepatic steatosis is histologically defined by lipid accumulation in at least 5% of hepatocytes [5]. Histological steatosis is conventionally graded as S0, S1, S2, or S3 according to the proportion of hepatocytes containing macrovesicular fat [6]. In the present study, S ≥ 2, corresponding to steatosis involving at least 34% of hepatocytes, was selected as the primary outcome because it represents a substantial hepatic fat burden and has been used as an analytical threshold in histological and non-invasive steatosis studies [6,7]. Although fibrosis is more directly associated with long-term liver-related outcomes than steatosis grade alone [8,9], identifying S ≥ 2 steatosis may help identify a subgroup with a more substantial hepatic fat burden and may therefore be useful for risk enrichment and triage for further liver-focused assessment.

The controlled attenuation parameter (CAP), obtained using vibration-controlled transient elastography, is widely used for the non-invasive assessment of hepatic steatosis [10]. However, its performance may be influenced by obesity-related body habitus, probe selection, and other clinical factors [11]. Recent biopsy-based studies have highlighted population-specific limitations of CAP, including discrepancies with histological steatosis assessment in bariatric populations and the potential need for obesity-specific CAP thresholds [12,13]. Nevertheless, refinement of CAP thresholds does not address whether routinely available biochemical markers provide additional predictive information beyond CAP itself. Alanine aminotransferase (ALT) is a routinely available marker associated with hepatocellular injury, whereas glycated haemoglobin (HbA1c) reflects longer-term glycaemic exposure [14,15]. Combining these markers with CAP may therefore integrate complementary information on hepatic fat accumulation, hepatocellular injury, and longer-term glycaemic exposure.

In this study, we examined whether adding ALT and HbA1c to CAP provides additional predictive information for identifying biopsy-confirmed moderate-to-severe hepatic steatosis (S ≥ 2) in bariatric surgery candidates. We developed and internally validated a parsimonious CAP–ALT–HbA1c model and evaluated its discrimination, calibration, prediction error, and net benefit relative to CAP alone and descriptively compared its discrimination with commonly used metabolic indices. The intended role of the model was to complement CAP as an adjunctive tool for preoperative risk stratification and clinical triage by helping identify patients with a higher probability of S ≥ 2 steatosis who may warrant further liver-focused assessment.

## 2. Materials and Methods

### 2.1. Study Design and Participants

This retrospective single-centre diagnostic prediction study included consecutive patients with obesity who underwent bariatric surgery at the Department of Hepatobiliary and Pancreatic Surgery and the Bariatric and Metabolic Diseases Surgery Center of Zhongnan Hospital of Wuhan University between January 2019 and December 2023. Patients were eligible if preoperative CAP measurements, laboratory data, and intraoperative liver biopsy results were available. Eligibility for bariatric surgery was determined according to the clinical standards in effect at the time of surgery; for consistency, all included patients were retrospectively confirmed to meet the 2022 ASMBS/IFSO indications [16].

Exclusion criteria were alcohol consumption exceeding 30 g/day in men or 20 g/day in women, viral hepatitis, autoimmune liver disease, drug-induced liver injury, other secondary causes of hepatic steatosis, severe systemic illness, and malignancy [17]. Transient elastography and biochemical assessments were performed within two weeks before surgery, and hepatic steatosis was assessed using intraoperative wedge liver biopsy. No missing values were present for the primary outcome or any candidate predictor used in model development.

The study was approved by the Medical Ethics Committee of Zhongnan Hospital of Wuhan University (No. 2019021) and was conducted in accordance with the Declaration of Helsinki and relevant institutional guidelines. All participants provided preoperative written informed consent for the use of anonymized clinical data for research purposes.

### 2.2. Clinical and Laboratory Assessments

Anthropometric and clinical measurements were obtained at the time of transient elastography. Central obesity was defined as waist circumference (WC) ≥ 90 cm in men or ≥ 80 cm in women [18]. The blood samples were collected after an overnight fast of at least 8 h, within three days prior to surgery. Laboratory analyses included alanine aminotransferase (ALT), aspartate aminotransferase (AST), gamma-glutamyl transferase (GGT), fasting plasma glucose (FPG), fasting insulin, total cholesterol (TC), triglycerides (TG), high-density lipoprotein cholesterol (HDL-C), low-density lipoprotein cholesterol (LDL-C), and glycated haemoglobin A1c (HbA1c). Homeostasis model assessment (HOMA-IR) was used to estimate insulin resistance, calculated as fasting insulin (µU/mL) × fasting glucose (mmol/L)/22.5 [19]. Type 2 diabetes mellitus (T2DM) and impaired glucose tolerance (IGT) were defined according to established diagnostic criteria or documented clinical diagnoses.

### 2.3. Liver Histology

Wedge liver biopsies were obtained intraoperatively under direct visualisation during bariatric surgery. Adequate biopsy specimens were defined as samples with a length ≥ 15 mm and ≥6 complete portal tracts [20]. Hepatic steatosis was graded according to the proportion of hepatocytes containing macrovesicular fat as S0 (<5%), S1 (5–33%), S2 (34–66%), or S3 (≥67%), and fibrosis stage, obtained from the formal final pathology reports, was categorized from F0 to F4 according to established histological scoring systems [6,21]. The primary histological outcome was prespecified as moderate-to-severe steatosis, defined as S ≥ 2 [22]. Advanced fibrosis was defined as F ≥ 3 [23].

### 2.4. Transient Elastography

Transient elastography was performed using the FibroScan 502 Touch system (Echosens, Paris, France) by trained operators before surgery. At least 10 valid measurements were obtained, and the median CAP and liver stiffness measurement (LSM) values, expressed in dB/m and kPa, respectively, were recorded for analysis. CAP measurement variability was assessed using the interquartile range, and a CAP-IQR/median ratio < 0.30 was considered reliable [24,25]. The XL probe was used in all participants because it is designed for individuals with increased body habitus and permits measurements at greater tissue depth [26,27].

### 2.5. Statistical Analysis and Model Development

Continuous variables were presented as mean ± standard deviation, and categorical variables as frequencies and percentages. Between-group comparisons were performed using independent-samples *t*-tests for continuous variables and the χ^2^ test or Fisher’s exact test for categorical variables, as appropriate. Comparisons across histological steatosis grades were performed using one-way analysis of variance. Moderate-to-severe steatosis (S ≥ 2) was prespecified as the primary outcome. Exploratory analyses were also performed at the S ≥ 1 and S ≥ 3 thresholds.

CAP and candidate demographic, anthropometric, biochemical, and metabolic predictors were initially evaluated using univariable logistic regression (Supplementary Table S5). Least absolute shrinkage and selection operator (LASSO) regression with ten-fold cross-validation was performed as a complementary screening procedure, with λ_min used to identify predictors with non-zero coefficients. LASSO findings were considered together with clinical relevance, redundancy among predictors, routine availability, and model parsimony. CAP, ALT, GGT, HbA1c, age, and WC were entered simultaneously into the candidate multivariable logistic regression model. Pairwise correlations among candidate predictors were assessed using Spearman correlation coefficients to evaluate predictor redundancy. The final model was selected according to adjusted associations, clinical interpretability, and parsimony. Variance inflation factors were calculated to assess multicollinearity among the final predictors. Model discrimination was quantified using the area under the receiver operating characteristic curve (AUC) with 95% confidence intervals. CAP was modelled as a continuous predictor in the CAP-only comparator. The AUCs of the CAP-only and CAP–ALT–HbA1c models were compared using the paired DeLong test. The Youden index was used to identify an exploratory threshold, at which sensitivity, specificity, positive predictive value, and negative predictive value were calculated. Overall prediction error was quantified using the Brier score.

Internal validation of the fixed final model was performed using 1000 bootstrap resamples. Bootstrap resampling was used to assess coefficient stability, estimate optimism in model discrimination, and calculate the optimism-corrected AUC. Calibration was assessed using a bootstrap bias-corrected calibration curve, and the mean absolute calibration error was reported. Apparent calibration intercept, calibration slope, and the Hosmer–Lemeshow goodness-of-fit test were reported descriptively.

Decision curve analysis was used to compare the net benefit of the CAP–ALT–HbA1c model, CAP alone, and the treat-all and treat-none strategies across threshold probabilities from 0.05 to 0.80. The clinical action associated with exceeding a threshold was defined as referral for additional liver-focused assessment. Sensitivity analyses included replacing HbA1c with HOMA-IR, additionally adjusting the final model for T2DM/IGT status, sex, or both, and separately adding age or AST to evaluate their incremental contributions. Additional sensitivity analyses separately extended the fixed final model with GGT, natural-log-transformed GGT, LSM, or histological fibrosis stage, and repeated the primary analysis after exclusion of participants with F ≥ 3 or F ≥ 2 fibrosis. For fixed-model extensions, model fit was assessed using likelihood-ratio tests and Akaike information criterion (AIC), with AUCs compared by paired DeLong tests when models were fitted to the same participants. Analyses were performed using SPSS version 29.0 and R version 4.3.2. The primary analyses were based on the 320 participants with complete CAP, laboratory, and histological data. All tests were two-sided, and *p* < 0.05 was considered statistically significant. A schematic overview of the study workflow is provided in Figure 1.

## 3. Results

### 3.1. Study Population and Baseline Characteristics

A total of 320 bariatric surgery candidates with complete liver biopsy, CAP, and laboratory data were included in the analysis. The mean age was 31.95 ± 7.47 years, 90 patients (28.1%) were male, and the mean BMI was 38.69 ± 5.54 kg/m^2^. The mean CAP and LSM values were 335.99 ± 23.39 dB/m and 12.22 ± 5.65 kPa, respectively, and 205 patients (64.1%) had T2DM or IGT. Histologically, 12 patients (3.8%) had S0, 126 (39.4%) had S1, 121 (37.8%) had S2, and 61 (19.1%) had S3 steatosis. Fibrosis was predominantly F0–F1, with advanced fibrosis (F ≥ 3) present in 11 patients (3.4%) (Supplementary Table S1).

For the primary comparison, 182 patients (56.9%) had moderate-to-severe steatosis (S ≥ 2), whereas 138 (43.1%) had S < 2 steatosis. Compared with patients with S < 2, those with S ≥ 2 were more frequently male (34.1% vs. 20.3%, *p* < 0.01) and younger (31.08 ± 7.00 vs. 33.10 ± 7.94 years, *p* = 0.02). The S ≥ 2 group also had higher BMI, WC, CAP and LSM values, as well as higher ALT, AST and GGT levels (all *p* < 0.01). In addition, patients with S ≥ 2 had lower HDL-C and higher FPG, HbA1c, insulin, and HOMA-IR levels, as well as a higher prevalence of T2DM or IGT (70.9% vs. 55.1%, *p* < 0.01). TG, LDL-C, uric acid, TC, and the prevalence of hypertension did not differ significantly between the two groups (Table 1).

In exploratory comparisons, patients with S ≥ 1 had higher CAP and LSM values than those with S0, and patients with S3 had higher CAP and LSM values than those with S ≤ 2 (Supplementary Tables S2 and S3). CAP values increased progressively across histological steatosis grades, from 315.08 ± 16.99 dB/m in S0 to 345.62 ± 26.64 dB/m in S3 (overall *p* < 0.001; Supplementary Table S4 and Figure S1).

### 3.2. Diagnostic Performance of CAP Across Histological Steatosis Thresholds

Using liver biopsy as the reference standard, CAP showed an AUC of 0.795 for detecting any steatosis (S ≥ 1), with a Youden index-derived cutoff of 319.5 dB/m. The AUC was 0.671 for moderate-to-severe steatosis (S ≥ 2) and 0.629 for severe steatosis (S ≥ 3), with corresponding cutoffs of 324.5 dB/m and 346.5 dB/m, respectively (Table 2 and Supplementary Figure S2). For the primary outcome of S ≥ 2 steatosis, CAP alone showed a sensitivity of 81.3%, specificity of 47.1%, positive predictive value (PPV) of 67.0%, and negative predictive value (NPV) of 65.7%.

### 3.3. Development of the CAP–ALT–HbA1c Model

Univariable logistic regression showed that CAP, ALT, AST, GGT, HbA1c, BMI, and WC were positively associated with S ≥ 2 steatosis, whereas HDL-C and age showed inverse associations (Supplementary Table S5). LASSO regression with ten-fold cross-validation was performed as a complementary variable-screening analysis. CAP, ALT, AST, GGT, HbA1c, TC, TG, HDL-C, WC, and age had non-zero coefficients, whereas LDL-C and BMI were shrunk to zero (Supplementary Table S6).

Because ALT and AST were strongly correlated (Spearman *r* = 0.92; Supplementary Figure S3), AST was not entered together with ALT in the candidate multivariable model to reduce redundancy. Based on the complementary screening results together with clinical relevance, routine availability, and model parsimony, CAP, ALT, GGT, HbA1c, age, and WC were entered simultaneously into the candidate multivariable logistic regression model. CAP, ALT, and HbA1c remained statistically significant after adjustment, whereas GGT, age, and WC were not (Supplementary Table S7). A parsimonious three-variable model comprising CAP, ALT, and HbA1c was therefore retained as the final model.

The estimated regression coefficients were 0.025 for CAP, 0.015 for ALT, and 0.258 for HbA1c. The final logistic regression equation was:

logit(*P*) = −10.474 + 0.025 × CAP (dB/m) + 0.015 × ALT (U/L) + 0.258 × HbA1c (%).

The corresponding ORs were 1.025 for CAP, 1.015 for ALT, and 1.294 for HbA1c, with bootstrap-derived 95% CIs shown in Table 3.

### 3.4. Incremental Discrimination and Internal Validation

The CAP–ALT–HbA1c model showed higher discrimination than CAP alone for detecting S ≥ 2 steatosis, with an AUC of 0.768 (95% CI 0.717–0.819) compared with 0.671 (95% CI 0.612–0.731) for CAP alone. The AUC difference was 0.096 (95% CI 0.049–0.144; paired DeLong *p* < 0.001; Figure 2 and Table 4).

At the Youden index-derived probability threshold of 0.6546, the CAP–ALT–HbA1c model showed a sensitivity of 53.8%, specificity of 86.2%, PPV of 83.8%, and NPV of 58.6%. The Brier score was lower for the combined model than for CAP alone (0.1941 vs. 0.2234), indicating lower overall prediction error (Table 4).

Internal validation using 1000 bootstrap resamples yielded an apparent AUC of 0.768, mean optimism of 0.007, and an optimism-corrected AUC of 0.760. The apparent and bootstrap bias-corrected calibration curves were closely aligned and remained near the ideal line across most of the predicted probability range. The mean absolute calibration error was 0.017, indicating close agreement between predicted and observed probabilities in the development cohort (Figure 3).

### 3.5. Model Diagnostics, Decision Curve Analysis, and Sensitivity Analyses

No substantial multicollinearity was observed among the final predictors, with variance inflation factor values of 1.037 for CAP, 1.032 for HbA1c, and 1.049 for ALT (Supplementary Table S8). Apparent calibration estimates and the Hosmer–Lemeshow goodness-of-fit result are provided in Supplementary Table S9.

Decision curve analysis showed similar net benefit for the CAP–ALT–HbA1c model and CAP alone at lower threshold probabilities. From approximately 0.30 to 0.80, the combined model generally provided higher net benefit than CAP alone, with greater separation at higher thresholds (Supplementary Figure S4).

Discrimination remained similar when HbA1c was replaced by HOMA-IR and when the primary model was additionally adjusted for T2DM/IGT status, sex, or both. The corresponding AUCs were 0.762, 0.772, 0.770, and 0.772, respectively (Supplementary Table S10). Adding age yielded an AUC of 0.767 (95% CI 0.716–0.819) and a Brier score of 0.1942, without improving discrimination or model fit compared with the primary model (paired DeLong *p* = 0.921; likelihood ratio test *p* = 0.384). Adding AST yielded an AUC of 0.766 (95% CI 0.715–0.818) and a Brier score of 0.1946, with paired DeLong *p* = 0.492 and likelihood ratio test *p* = 0.530. The Akaike information criterion was lower for the primary model than for the age- or AST-adjusted models (381.21 vs. 382.46 and 382.82, respectively; Supplementary Table S11).

Adding raw GGT did not significantly improve model fit or discrimination. Although ln(GGT) was independently associated with S ≥ 2 steatosis and improved model fit (likelihood ratio test *p* = 0.017), discrimination remained essentially unchanged (AUC 0.766 vs. 0.768; paired DeLong *p* = 0.847; Supplementary Table S12). Neither LSM nor histological fibrosis stage materially improved discrimination when added separately to the primary model (AUCs 0.767 and 0.769, respectively). Model performance remained similar after excluding participants with F ≥ 3 fibrosis (AUC 0.767) or F ≥ 2 fibrosis (AUC 0.763; Supplementary Table S13).

Overall, model discrimination remained broadly similar across the alternative and additionally adjusted specifications.

### 3.6. Comparison with Conventional Metabolic Indices

The diagnostic performance of the CAP–ALT–HbA1c model was further compared with several established metabolic indices, including HSI [28], NLFS [29], ZJU [30], TyG [31], LAP [32], and VAI [33]. Among these conventional indices, NLFS showed the highest AUC for detecting S ≥ 2 steatosis (AUC 0.712; 95% CI 0.655–0.769), followed by HSI (AUC 0.664; 95% CI 0.604–0.724). The remaining indices showed lower AUCs, ranging from 0.539 to 0.634 (Table 5 and Supplementary Figure S5). All evaluated conventional metabolic indices showed numerically lower AUCs than the CAP–ALT–HbA1c model.

## 4. Discussion

In this biopsy-referenced cohort of bariatric surgery candidates, we developed and internally validated a parsimonious CAP–ALT–HbA1c model for identifying moderate-to-severe hepatic steatosis (S ≥ 2). Adding ALT and HbA1c to CAP was associated with higher discrimination than CAP alone, with an AUC difference of 0.096. The combined model also showed lower overall prediction error. Bootstrap internal validation indicated limited optimism, with an optimism-corrected AUC of 0.760 and a mean absolute calibration error of 0.017. Model discrimination was largely preserved across alternative and additionally adjusted specifications, including the GGT-, LSM-, and fibrosis-related sensitivity analyses. Decision curve analysis suggested modest incremental net benefit over CAP alone across much of the evaluated threshold range. Collectively, these findings indicate that routinely available biochemical variables may provide complementary predictive information when considered alongside CAP in bariatric surgery candidates.

Early biopsy-referenced studies established CAP as a non-invasive measure associated with histological steatosis severity [34,35,36]. However, subsequent evidence has shown that its diagnostic performance and optimal cutoffs vary across steatosis thresholds and patient populations [7,11]. An individual patient data meta-analysis found that CAP values were influenced by disease aetiology, diabetes, BMI, AST, and sex, and that optimal cutoffs differed substantially across aetiologies [11]. Obesity-specific biopsy studies have also reported suboptimal performance of conventional CAP cutoffs and suggested that higher or recalibrated thresholds may be required in bariatric and other populations with obesity [13,22]. In the present cohort, CAP discrimination was higher for detecting any steatosis than for the S ≥ 2 and S ≥ 3 thresholds. Although population-specific recalibration may improve CAP interpretation, threshold adjustment alone does not determine whether additional routinely available clinical information contributes incremental predictive value. Our study therefore extended beyond CAP cutoff optimisation by evaluating ALT and HbA1c as complementary predictors of biopsy-confirmed S ≥ 2 steatosis.

The inclusion of ALT and HbA1c is clinically and biologically plausible, although these variables should be interpreted as complementary predictors rather than direct mechanistic measures. Obesity-associated hepatic fat accumulation reflects increased fatty acid delivery and broader metabolic dysfunction [37,38]. HbA1c reflects longer-term glycaemic exposure [15], whereas ALT is a routinely available biochemical marker associated with hepatocellular injury [39]. Lipotoxic intermediates may contribute to hepatocellular stress and injury in steatotic liver disease [40]. The CAP–ALT–HbA1c model may therefore integrate information from three clinically distinct domains: imaging-derived ultrasound attenuation related to hepatic fat, longer-term glycaemic exposure, and liver injury-related biochemistry. This interpretation is consistent with previous bariatric biopsy cohorts in which elevated liver enzymes and dysglycaemia, including higher HbA1c, were associated with more advanced histological liver disease [41].

Our findings regarding GGT are also broadly consistent with previous bariatric biopsy studies. In a cohort of 1000 patients undergoing intraoperative wedge biopsy, GGT was associated with severe steatosis and was higher in patients with bridging fibrosis, while another bariatric biopsy study found elevated GGT to be associated with NASH but not retained as an independent risk factor after multivariable assessment [42,43]. Longitudinal paired-biopsy data have also shown that reductions in GGT accompany histological improvement after weight loss [44]. These observations are consistent with our finding that GGT was associated with S ≥ 2 steatosis in univariable analysis but provided limited independent information after accounting for CAP, ALT, HbA1c, age, and WC. Although log-transformed GGT improved model fit, it did not materially improve discrimination, suggesting that GGT may reflect relevant hepatometabolic burden without contributing sufficient additional discriminatory information to justify increasing model complexity. The similar discrimination of the CAP–ALT–HOMA-IR model and the models additionally adjusted for dysglycaemia status suggests that model performance was not driven solely by the particular glycaemic marker used. HbA1c may nevertheless offer practical advantages because it is routinely measured and does not require fasting insulin assessment. Furthermore, adding AST or age did not materially improve discrimination or overall prediction error, supporting retention of the more parsimonious three-variable specification in this cohort.

Comparison with conventional metabolic indices provided descriptive context for the performance of the combined model. Among the evaluated indices, NLFS showed the highest discrimination, although its AUC remained numerically lower than that of the CAP–ALT–HbA1c model. This difference may partly reflect the incorporation of an imaging-derived measure into the proposed model, whereas most conventional indices rely predominantly on anthropometric and laboratory variables. A recent study developed a nomogram using BMI, TG, adiponectin, and chemerin in patients with abnormal glucose metabolism, but severe steatosis was defined using a CAP threshold rather than liver histology [45]. That study and the present analysis therefore address related but distinct prediction settings. Our study used intraoperative liver biopsy as the reference standard, focused specifically on bariatric surgery candidates, and relied on routinely available predictors. Nevertheless, comparisons with conventional metabolic indices were descriptive, and the numerical differences in AUC should not be interpreted as statistically confirmed superiority in the absence of formal pairwise tests.

Fibrosis represents a distinct prognostic dimension of MASLD. Meta-analyses of biopsy-confirmed NAFLD have consistently shown progressively higher all-cause and liver-related mortality with advancing fibrosis stage [46,47], and current guidelines recommend stepwise non-invasive assessment, including transient elastography, for identifying patients at risk of advanced fibrosis [2]. Importantly, significant fibrosis is relatively uncommon in large bariatric surgical cohorts; in a series of 2557 routine intraoperative biopsies, 7.8% of patients had stage ≥ 2 fibrosis [48], closely resembling the 7.5% prevalence of F ≥ 2 in our cohort. In the present study, neither LSM nor histological fibrosis stage materially improved discrimination, and discrimination was largely preserved after excluding participants with F ≥ 3 or F ≥ 2 fibrosis. These findings reduce the likelihood that the performance of the steatosis model was driven primarily by the small subgroup with more advanced fibrosis, although validation in populations with a broader fibrosis spectrum remains warranted.

From a clinical perspective, the CAP–ALT–HbA1c model may serve as an adjunctive preoperative risk-stratification tool rather than a standalone diagnostic test. Although the combined model showed improved discrimination compared with CAP alone, its operating characteristics at the internally derived Youden threshold were characterised by relatively high specificity and positive predictive value but modest sensitivity (53.8%) and negative predictive value. This operating profile suggests that it may be more suitable for enriching a group with a high probability of S ≥ 2 steatosis than for ruling out disease. If externally validated, patients with a higher predicted probability of S ≥ 2 steatosis could be considered for more detailed liver-focused assessment, including quantitative imaging such as MRI-PDFF where available and clinically appropriate [2,5,49]. The decision curve findings also suggested potential incremental decision value over CAP alone, particularly at moderate-to-high threshold probabilities. However, decision curve analysis does not establish improvement in patient outcomes, and its interpretation depends on the clinical action assigned to a given threshold. Moreover, positive and negative predictive values are prevalence-dependent, and the high prevalence of S ≥ 2 steatosis in this bariatric cohort limits direct extrapolation to lower-prevalence populations. The probability threshold identified in this study should therefore be regarded as exploratory. External validation should apply the fixed model equation to independent cohorts and reassess discrimination, calibration, overall prediction error, and clinical utility; recalibration or selection of a clinically appropriate decision threshold may be required before implementation in populations with different case mix or disease prevalence.

Several features strengthen the present study, including the use of liver biopsy as the reference standard, consecutive inclusion of bariatric surgery candidates, direct comparison with CAP alone, evaluation of overall prediction error and calibration, bootstrap internal validation, decision curve analysis, and targeted sensitivity analyses addressing alternative metabolic markers, initially screened variables, liver stiffness, and histological fibrosis. The model also contains only three routinely available predictors, which facilitates interpretation and subsequent external validation.

Several limitations should nevertheless be acknowledged. First, this was a retrospective, single-centre study involving a relatively young bariatric surgical population, which may limit generalisability to older individuals and non-surgical populations with obesity. Although age did not materially improve model performance in sensitivity analysis, this within-cohort finding does not replace validation in older populations. Second, advanced fibrosis was infrequent, limiting conclusions regarding patients with substantial fibrotic or cirrhotic disease despite the stability observed in fibrosis-related sensitivity analyses. Although intraoperative wedge biopsy provided a histological reference standard, sampling variability inherent to liver biopsy cannot be completely excluded [50]. Previous bariatric studies comparing wedge and core biopsies have also reported variability in individual histological components, particularly fibrosis stage [51]. Third, incomplete information on glucose-lowering and lipid-lowering therapies may have contributed to residual confounding. Finally, although bootstrap resampling provided internal validation, the model has not yet undergone external validation. Independent validation in broader, preferably multicentre cohorts is therefore required before clinical implementation.

## 5. Conclusions

In this biopsy-referenced cohort of bariatric surgery candidates, the internally validated CAP–ALT–HbA1c model showed higher discrimination and lower overall prediction error than CAP alone for identifying moderate-to-severe hepatic steatosis. The parsimonious model may complement CAP as an adjunctive tool for preoperative risk stratification by helping identify patients with a higher probability of biopsy-confirmed S ≥ 2 steatosis. External validation and assessment of clinically appropriate decision thresholds are required before routine clinical implementation.

## Figures and Tables

**Figure 1:**
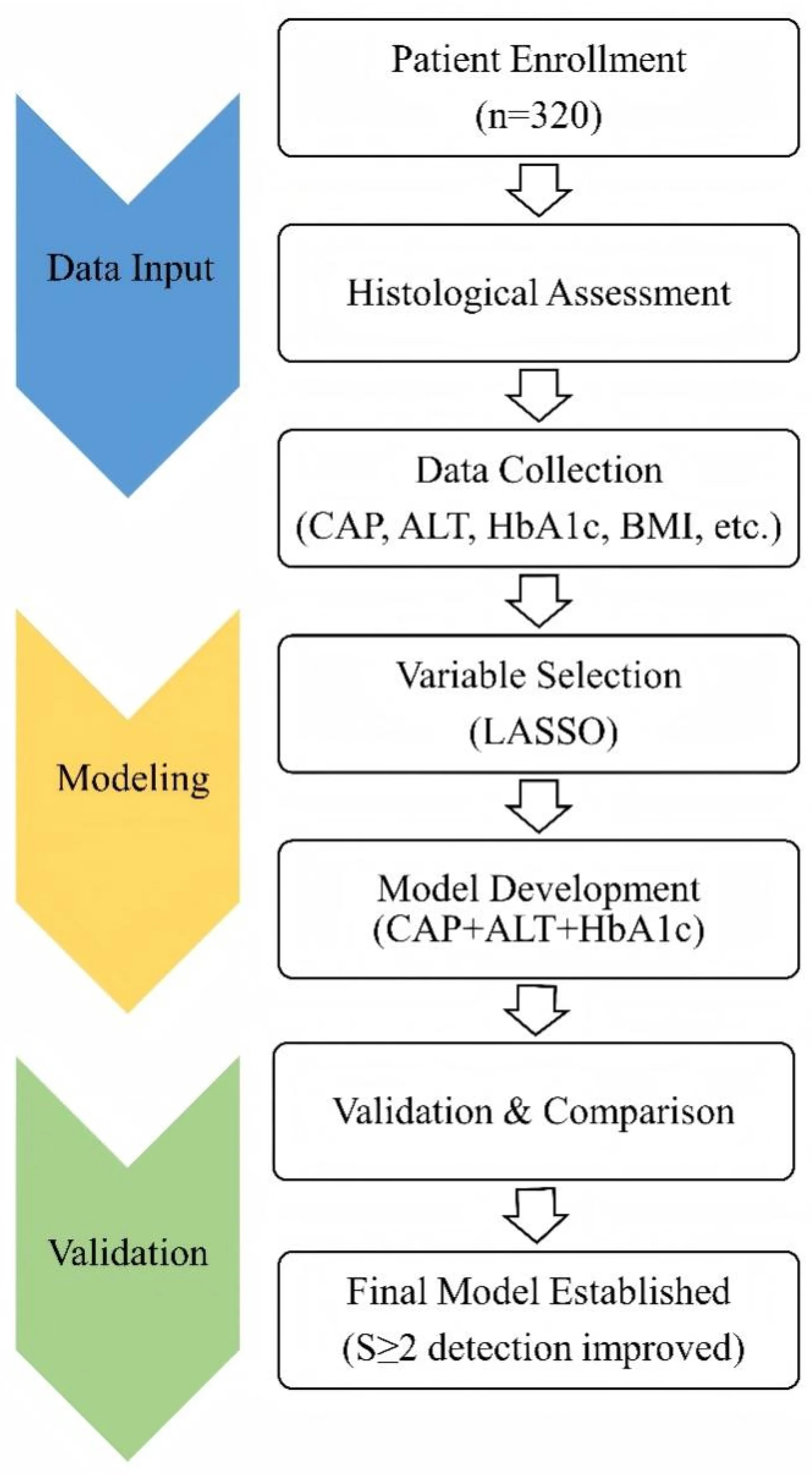
Study workflow for CAP–ALT–HbA1c model development and evaluation. The workflow includes three stages: (1) patient recruitment and data collection, (2) variable selection and model construction, and (3) model performance evaluation and clinical utility assessment.

**Figure 2:**
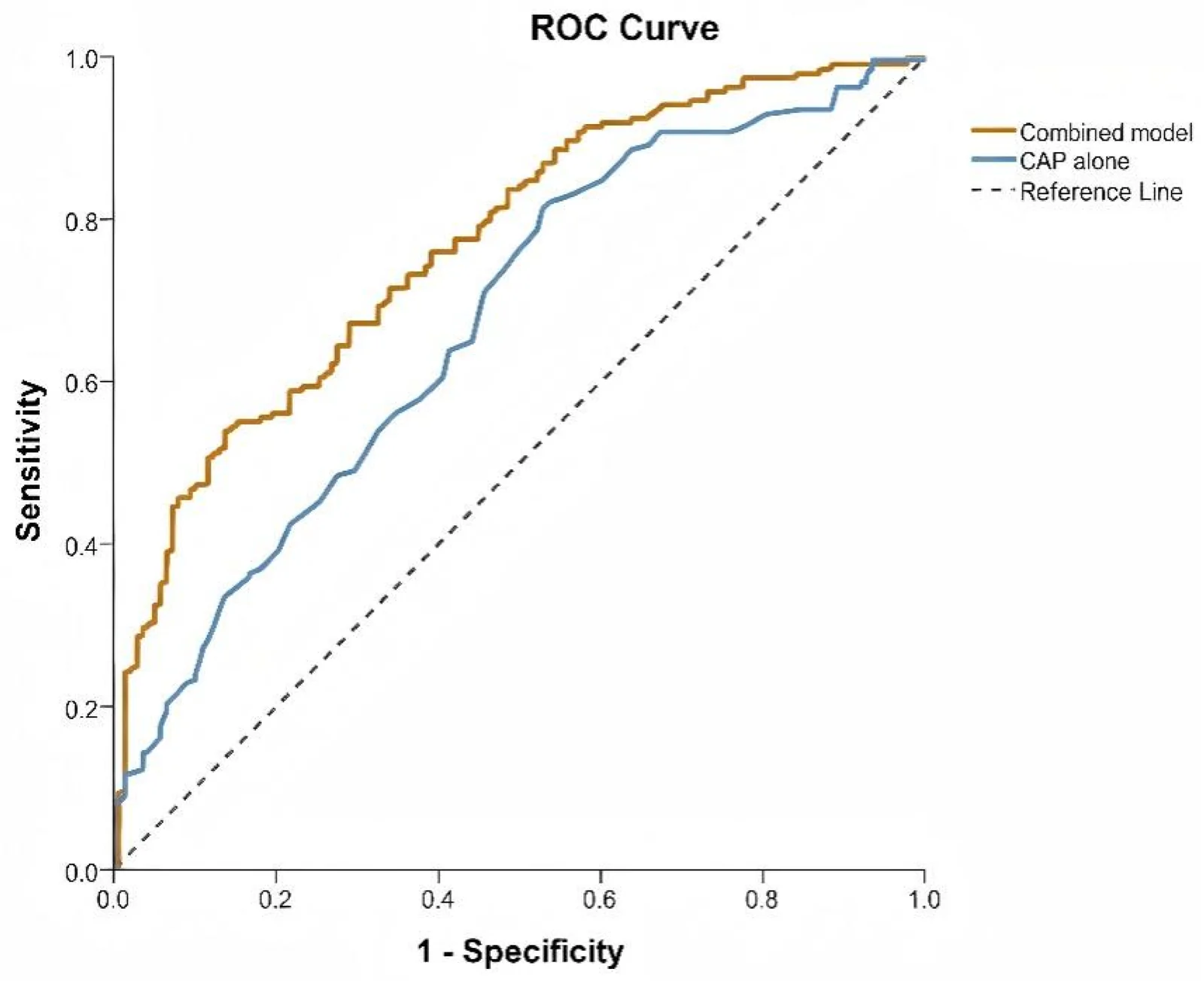
ROC curves for the CAP–ALT–HbA1c model (orange) and CAP alone (blue) in detecting moderate-to-severe steatosis (S ≥ 2) using liver biopsy as the reference. The CAP–ALT–HbA1c model demonstrated improved discrimination compared with CAP alone (AUC 0.768 vs. 0.671, DeLong test *p* < 0.001).

**Figure 3:**
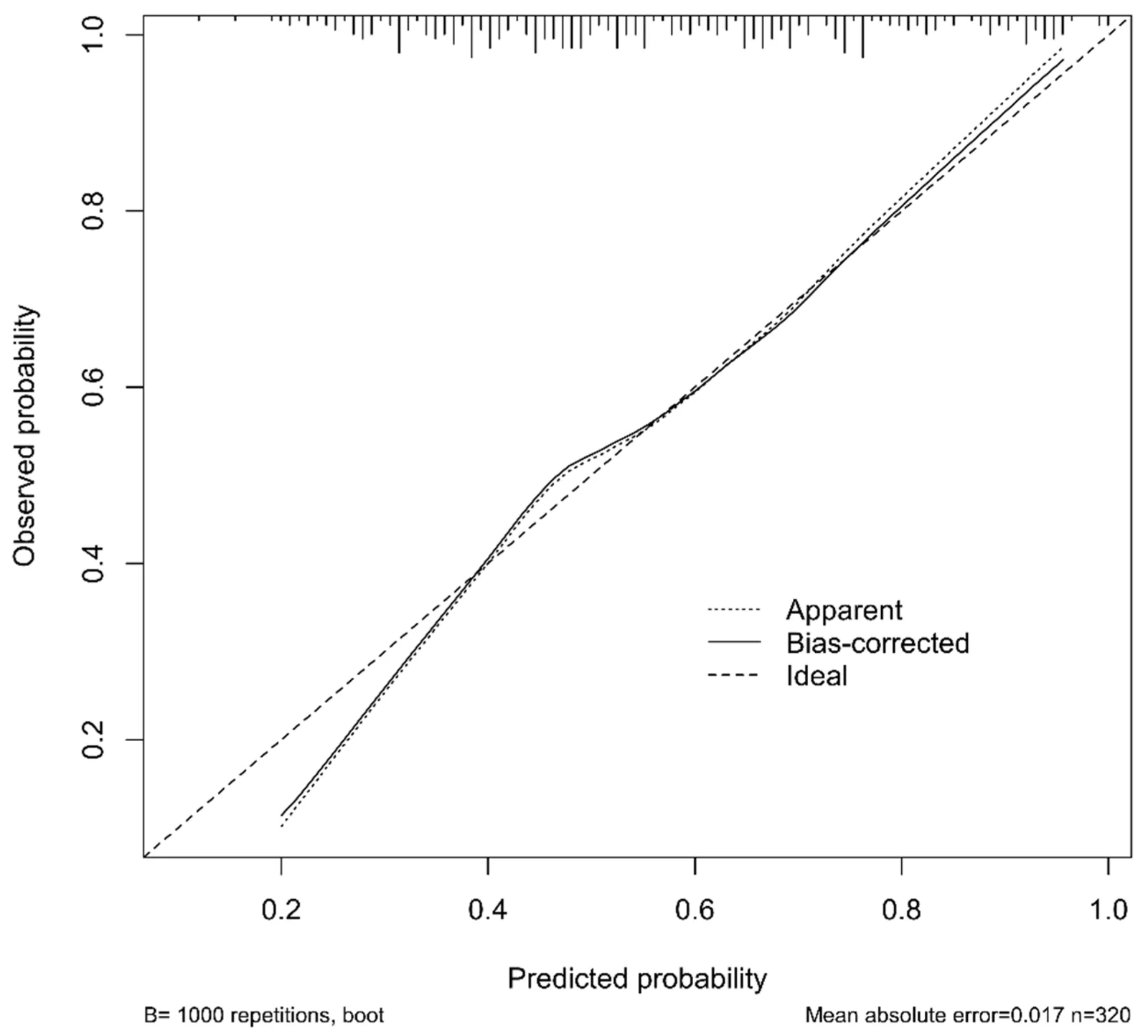
Bootstrap bias-corrected calibration plot of the CAP–ALT–HbA1c model for biopsy-confirmed moderate-to-severe hepatic steatosis (S ≥ 2). The x-axis represents the predicted probability of S ≥ 2 steatosis, and the y-axis represents the corresponding observed probability. The dashed line indicates perfect calibration, the dotted line represents apparent calibration in the development cohort, and the solid line represents bootstrap bias-corrected calibration based on 1000 resamples. The apparent and bias-corrected curves were closely aligned, with a mean absolute calibration error of 0.017. Rug marks at the top indicate the distribution of predicted probabilities among the 320 participants.

**Table 1:** Baseline characteristics stratified by histological steatosis severity (S < 2 vs. S ≥ 2).

Variable	S < 2 (*n* = 138)	S ≥ 2 (*n* = 182)	*p* Value
Demographics			
Male, *n* (%)	28 (20.3)	62 (34.1)	<0.01
Age, years	33.10 ± 7.94	31.08 ± 7.00	0.02
Anthropometrics			
BMI, kg/m^2^	37.64 ± 4.96	39.48 ± 5.83	<0.01
WC, cm	116.97 ± 12.78	121.59 ± 14.45	< 0.01
Imaging markers			
CAP, dB/m	328.10 ± 19.57	341.98 ± 24.31	<0.01
LSM, kPa	10.88 ± 4.79	13.24 ± 6.04	<0.01
Liver biochemistry			
ALT, U/L	43.82 ± 57.64	77.19 ± 51.69	<0.01
AST, U/L	29.11 ± 24.72	48.56 ± 36.15	<0.01
GGT, U/L	39.86 ± 28.81	61.10 ± 45.70	<0.01
Metabolic parameters			
Uric acid, μmol/L	445.20 ± 118.47	468.83 ± 123.39	0.09
TC, mmol/L	5.01 ± 1.01	4.89 ± 0.87	0.23
TG, mmol/L	2.04 ± 1.60	2.10 ± 1.21	0.71
HDL-C, mmol/L	1.10 ± 0.23	1.03 ± 0.18	<0.01
LDL-C, mmol/L	3.26 ± 0.91	3.21 ± 0.75	0.56
FPG, mmol/L	5.58 ± 2.32	6.18 ± 2.32	0.02
HbA1c, %	5.91 ± 1.16	6.45 ± 1.47	<0.01
Insulin, μU/mL	25.47 ± 13.98	30.27 ± 14.99	<0.01
HOMA-IR	6.22 ± 4.24	8.03 ± 4.13	<0.01
Comorbidities			
IGT/T2DM, *n* (%)	76 (55.1)	129 (70.9)	<0.01
Hypertension, *n* (%)	32 (23.2)	32 (17.6)	0.21

Continuous variables are expressed as mean ± SD; categorical variables are presented as number (percentage). *p* values were calculated using independent-samples *t*-tests or χ^2^ test, as appropriate. *p* < 0.05 was considered statistically significant. Abbreviations: BMI, body mass index; WC, waist circumference; CAP, controlled attenuation parameter; LSM, liver stiffness measurement; ALT, alanine aminotransferase; AST, aspartate aminotransferase; GGT, gamma-glutamyl transferase; TC, total cholesterol; TG, triglycerides; HDL-C, high-density lipoprotein cholesterol; LDL-C, low-density lipoprotein cholesterol; FPG, fasting plasma glucose; HOMA-IR, homeostasis model assessment of insulin resistance; HbA1c, glycated haemoglobin A1c; T2DM, type 2 diabetes mellitus; IGT, impaired glucose tolerance.

**Table 2:** Diagnostic performance of CAP at different histological steatosis thresholds.

Outcome	AUC (95% CI)	Cutoff (dB/m)	Se (%)	Sp (%)	PPV (%)	NPV (%)
S ≥ 1	0.795 (0.647–0.943)	319.5	80.2	83.3	99.2	14.1
S ≥ 2	0.671 (0.612–0.731)	324.5	81.3	47.1	67.0	65.7
S ≥ 3	0.629 (0.551–0.707)	346.5	41.0	78.8	31.3	85.0

Data are shown as area under the receiver operating characteristic curve (AUC) with 95% confidence intervals (CIs), optimal cutoff values determined by the Youden index. Abbreviations: Se, sensitivity; Sp, specificity; PPV, positive predictive value; NPV, negative predictive value.

**Table 3:** Final CAP–ALT–HbA1c logistic regression model for detecting biopsy-confirmed moderate-to-severe steatosis (S ≥ 2).

Variable	β (SE)	OR (Bootstrap 95% CI)	*p* Value
CAP (per dB/m)	0.025 (0.006)	1.025 (1.012–1.040)	< 0.001
ALT (per U/L)	0.015 (0.004)	1.015 (1.003–1.032)	< 0.001
HbA1c (per 1%)	0.258 (0.109)	1.294 (1.060–1.600)	0.018
Constant	−10.474 (2.200)	—	< 0.001

Data are presented as regression coefficients (β), standard errors (SE), odds ratios (ORs) with 95% confidence intervals (CIs). The 95% CIs for the ORs were derived from 1000 bootstrap resamples. Abbreviations: CAP, controlled attenuation parameter; ALT, alanine aminotransferase; HbA1c, glycated haemoglobin A1c; OR, odds ratio; CI, confidence interval; SE, standard error.

**Table 4:** Diagnostic performance of CAP alone and the CAP–ALT–HbA1c model for detecting moderate-to-severe steatosis (S ≥ 2).

Model	AUC	Decision Threshold	95% CI	Se (%)	Sp (%)	PPV (%)	NPV (%)	Brier Score
CAP alone	0.671	324.5 dB/m	0.612–0.731	81.3	47.1	67.0	65.7	0.2234
CAP–ALT–HbA1c model	0.768	0.6546	0.717–0.819	53.8	86.2	83.8	58.6	0.1941

Data are presented as area under the receiver operating characteristic curve (AUC) with 95% confidence intervals (CIs), sensitivity (Se), specificity (Sp), positive predictive value (PPV), negative predictive value (NPV), and Brier score. The cutoff for CAP alone is expressed in dB/m; the predicted probability for the CAP–ALT–HbA1c model was derived from logistic regression. Optimal thresholds were determined using the Youden index. The AUC difference between the CAP–ALT–HbA1c model and CAP alone was 0.096 (95% CI 0.049–0.144; paired DeLong *p* < 0.001). Abbreviations: CAP, controlled attenuation parameter; ALT, alanine aminotransferase; HbA1c, glycated haemoglobin A1c; AUC, area under the curve; CI, confidence interval; Se, sensitivity; Sp, specificity; PPV, positive predictive value; NPV, negative predictive value.

**Table 5:** Discrimination of conventional metabolic indices for detecting moderate-to-severe steatosis (S ≥ 2).

Model	AUC	Std. Error	95% CI
HSI	0.664	0.030	0.604–0.724
NLFS	0.712	0.029	0.655–0.769
ZJU	0.634	0.031	0.572–0.695
TyG	0.587	0.032	0.523–0.650
LAP	0.566	0.032	0.503–0.629
VAI	0.539	0.032	0.475–0.602

Data are presented as AUC with standard errors (Std. Error) and 95% confidence intervals (CI). Among all indices, the NAFLD liver fat score (NLFS) achieved the highest discrimination (AUC = 0.712), followed by the hepatic steatosis index (HSI, AUC = 0.664). Abbreviations: HSI, hepatic steatosis index; NLFS, NAFLD liver fat score; ZJU, Zhejiang University index; TyG, triglyceride-glucose index; LAP, lipid accumulation product; VAI, visceral adiposity index; AUC, area under the curve; CI, confidence interval.

## Data Availability

The datasets generated during and/or analysed during the current study are available from the corresponding author on reasonable request. The R code used for model development, receiver operating characteristic analysis, bootstrap internal validation, calibration assessment, and decision curve analysis is available from the corresponding authors on reasonable request.

## Supplementary Materials

The following supporting information can be downloaded at: https://www.mdpi.com/article/10.3390/jcm15197493/s1, Table S1: Baseline characteristics of the whole cohort (*n* = 320); Table S2: Baseline characteristics between patients with S = 0 and S ≥ 1; Table S3: Baseline characteristics between patients with S ≤ 2 and S3; Table S4: CAP values across histological steatosis grades in bariatric surgery candidates; Table S5: Univariable logistic regression analysis of selected candidate predictors for moderate-to-severe steatosis (S ≥ 2); Table S6: LASSO regression-selected candidate predictors for biopsy-confirmed moderate-to-severe hepatic steatosis (S ≥ 2); Table S7: Candidate multivariable logistic regression model for detecting moderate-to-severe steatosis (S ≥ 2); Table S8: Multicollinearity diagnostics for predictors included in the final CAP–ALT–HbA1c model; Table S9: Apparent calibration and overall prediction performance of the CAP–ALT–HbA1c model for detecting moderate-to-severe steatosis (S ≥ 2); Table S10: Sensitivity analyses of alternative and adjusted logistic regression models for biopsy-confirmed moderate-to-severe hepatic steatosis (S ≥ 2); Table S11: Targeted sensitivity analyses evaluating the incremental contributions of age and AST to the primary CAP–ALT–HbA1c model; Table S12: Additional sensitivity analyses evaluating the incremental contribution of GGT to the primary CAP–ALT–HbA1c model; Table S13: Histological fibrosis distribution and sensitivity analyses of liver stiffness and fibrosis in the CAP–ALT–HbA1c model; Figure S1: Distribution of CAP values across histological steatosis grades (S0 – S3); Figure S2: ROC curves of CAP for detecting histological steatosis at different thresholds; Figure S3: Spearman correlation heatmap among candidate predictors for multivariable modelling; Figure S4: Decision curve analysis of CAP alone and the CAP–ALT–HbA1c model; Figure S5: ROC curves of conventional metabolic indices for detecting biopsy-confirmed moderate-to-severe hepatic steatosis.
