# Supplementary material for: A CAP–ALT–HbA1c Model for Biopsy-Confirmed Moderate-to-Severe Hepatic Steatosis in Bariatric Surgery Candidates: Development and Internal Validation
Source: J Clin Med. 2026 Sep 26;15(19):7493. doi: 10.3390/jcm15197493 (PMC13666499; doi:10.3390/jcm15197493)
Supplement: Supplementary file 1 [file jcm-15-07493-s001.zip › jcm-4505526-supplementary.pdf]

Title:

A CAP–ALT–HbA1c Model for Biopsy-Confirmed Moderate-to-Severe Hepatic Steatosis in Bariatric Surgery Candidates: Development and Internal Validation

Authors:

Jingli Ding, Mengjie Hu, He Wang, Cheng Gong, Zixuan Mei, Wanshun Chun, Dawei Deng,

Zixuan Ma, Xiang Zhang, Qiu Zhao, Jianliang Zhou and Zhen Li

Supplementary Table S1. Baseline characteristics of the whole cohort (*n* = 320).

Variable	Mean ± SD or *n* (%)
**Demographics**	
Male, *n* (%)	90 (28.1)
Age, years	31.95 ± 7.47
**Anthropometrics**	
BMI, kg/m²	38.69 ± 5.54
WC, cm	119.60 ± 13.92
**Imaging markers**	
CAP, dB/m	335.99 ± 23.39
LSM, kPa	12.22 ± 5.65 kPa
**Liver biochemistry**	
ALT, U/L	62.80 ± 56.72
AST, U/L	40.17 ± 33.12
GGT, U/L	51.94 ± 40.65
**Metabolic parameters**	
Uric acid, μmol/L	458.64 ± 121.67
TC, mmol/L	4.94 ± 0.93
TG, mmol/L	2.07 ± 1.39
HDL-C, mmol/L	1.06 ± 0.20
LDL-C, mmol/L	3.24 ± 0.82
FPG, mmol/L	5.92 ± 2.33
HbA1c, %	6.22 ± 1.37
Insulin, μU/mL	28.20 ± 14.73
HOMA-IR	7.25 ± 4.26

**Histological characteristics**	
Steatosis grade, n (%) S0 / S1 / S2 / S3	12 (3.8) / 126 (39.4) / 121 (37.8) / 61 (19.1)
Fibrosis stage, n (%) F0 / F1 / F2 / F3 / F4	199 (62.2) / 97 (30.3) / 13 (4.1) / 10 (3.1) / 1 (0.3)
**Comorbidities**	
IGT/T2DM, *n* (%)	205 (64.1)
Hypertension, *n* (%)	64 (20.0)

Data are presented as mean ± standard deviation (SD) for continuous variables or number (percentage) for categorical variables. Abbreviations: BMI, body mass index; WC, waist circumference; CAP, controlled attenuation parameter; LSM, liver stiffnes s measurement; ALT, alanine aminotransferase; AST, aspartate aminotransferase; GG T, gamma-glutamyl transferase; HDL-C, high-density lipoprotein cholesterol; LDL-C, low-density lipoprotein cholesterol; TC, total cholesterol; TG, triglycerides; FPG, fast ing plasma glucose; HOMA-IR, homeostasis model assessment of insulin resistance; HbA1c, glycated haemoglobin A1c; T2DM, type 2 diabetes mellitus; IGT, impaired gl ucose tolerance.

Supplementary Table S2. Baseline characteristics between patients with S = 0 and S ≥ 1.

Variable	S = 0 (*n* = 12)	S ≥ 1 (*n* = 308)	*P* value
**Demographics**			
Male, *n* (%)	1 (8.3)	89 (28.9)	0.19
Age, years	32.67 ± 9.23	31.93 ± 7.41	0.79
**Anthropometrics**			
BMI, kg/m²	38.40 ± 5.92	38.70 ± 5.53	0.85
WC, cm	116.17 ± 15.21	119.73 ± 13.88	0.39
**Imaging markers**			
CAP, dB/m	315.08 ± 16.99	336.81 ± 23.25	<0.01
LSM, kPa	7.82 ± 2.09	12.39 ± 5.68	<0.01
**Liver biochemistry**			
ALT, U/L	20.25 ± 9.15	64.46 ± 57.15	<0.01
AST, U/L	19.67 ± 4.89	40.97 ± 33.49	<0.01
GGT , U/L	24.67 ± 6.68	53.00 ± 41.05	<0.01
**Metabolic parameters**			
Uric acid, µmol/L	437.55 ± 122.17	459.46 ± 121.78	0.54
TC, mmol/L	5.00 ± 1.16	4.94 ± 0.92	0.82
TG, mmol/L	1.11 ± 0.30	2.11 ± 1.40	0.02
HDL-C, mmol/L	1.27 ± 0.30	1.05 ± 0.19	0.03
LDL-C, mmol/L	3.06 ± 1.12	3.24 ± 0.81	0.44
FPG, mmol/L	4.63 ± 0.62	5.97 ± 2.36	<0.01
HbA1c, %	5.56 ± 0.49	6.24 ± 1.38	0.09
Insulin, µU/mL	19.33 ± 9.36	28.55 ± 14.81	0.03

HOMA-IR	4.06 ± 2.19	7.37 ± 4.27	<0.01
**Comorbidities**			
IGT/T2DM, *n* (%)	6 (50.0)	199 (64.6)	0.36
Hypertension, *n* (%)	2 (16.7)	62 (20.1)	1.00

Data are presented as mean ± standard deviation (SD) for continuous variables or number (percentage) for categorical variables. *P* values were calculated using independent-samples *t*-test for continuous variables or χ^2 test for categorical variables, as appropriate. Abbreviations: BMI, body mass index; WC, waist circumference; CAP, controlled attenuation parameter; LSM, liver stiffness measurement; ALT, alanine aminotransferase; AST, aspartate aminotransferase; GGT, gamma-glutamyl transferase; HDL-C, high-density lipoprotein cholesterol; LDL-C, low-density lipoprotein cholesterol; TC, total cholesterol; TG, triglycerides; FPG, fasting plasma glucose; HOMA-IR, homeostasis model assessment of insulin resistance; HbA1c, glycated haemoglobin A1c; T2DM, type 2 diabetes mellitus; IGT, impaired glucose tolerance.

Supplementary Table S3. Baseline characteristics between patients with S ≤ 2 and S3.

**Variable**	S ≤ 2 (*n* = 259)	S3 (*n* = 61)	*P* value
**Demographics**			
Male, *n* (%)	71 (27.4)	19 (31.1)	0.56
Age, years	32.46 ± 7.64	29.79 ± 6.31	0.01
**Anthropometrics**			
BMI, kg/m^2	38.32 ± 5.07	40.25 ± 7.02	0.04
WC, cm	118.64 ± 12.90	123.66 ± 17.14	0.04
**Imaging marker**			
CAP, dB/m	333.73 ± 22.01	345.62 ± 26.64	<0.01
LSM, kPa	11.75 ± 5.22	14.20 ± 6.91	<0.01
**Liver biochemistry**			
ALT, U/L	56.58 ± 55.87	89.23 ± 52.93	<0.01
AST, U/L	35.70 ± 28.75	59.16 ± 42.72	<0.01
GGT , U/L	47.08 ± 33.18	72.59 ± 59.31	<0.01
**Metabolic parameters**			
Uric acid, µmol/L	455.92 ± 120.97	470.18 ± 124.96	0.41
TC, mmol/L	4.98 ± 0.96	4.75 ± 0.81	0.08
TG, mmol/L	2.10 ± 1.48	1.95 ± 0.89	0.45
HDL-C, mmol/L	1.07 ± 0.21	1.03 ± 0.17	0.16
LDL-C, mmol/L	3.27 ± 0.83	3.11 ± 0.80	0.19
FPG, mmol/L	5.80 ± 2.27	6.44 ± 2.55	0.06
HbA1c, %	6.13 ± 1.27	6.60 ± 1.68	0.04
Insulin, µU/mL	27.11 ± 13.55	32.84 ± 18.39	0.03
HOMA-IR	6.83 ± 3.96	9.04 ± 5.03	<0.01

**Comorbidities**			
IGT/T2DM, *n* (%)	157 (60.6)	48 (78.7)	<0.01
Hypertension, *n* (%)	56 (21.6)	8 (13.1)	0.14

Data are presented as mean ± standard deviation (SD) for continuous variables or number (percentage) for categorical variables. *P* values were calculated using indepe ndent-samples *t*-test for continuous variables or χ^2 test for categorical variables, as app ropriate. Abbreviations: BMI, body mass index; WC, waist circumference; CAP, contr olled attenuation parameter; LSM, liver stiffness measurement; ALT, alanine aminotra nsferase; AST, aspartate aminotransferase; GGT, gamma-glutamyl transferase; HDL-C, high-density lipoprotein cholesterol; LDL-C, low-density lipoprotein cholesterol; T C, total cholesterol; TG, triglycerides; FPG, fasting plasma glucose; HOMA-IR, home ostasis model assessment of insulin resistance; HbA1c, glycated haemoglobin A1c; T 2DM, type 2 diabetes mellitus; IGT, impaired glucose tolerance.

Supplementary Table S4. CAP values across histological steatosis grades in bariatric surgery candidates

Steatosis grade	*n* (%)	CAP (mean ± SD, dB/m)
S0	12 (3.8)	315.08 ± 16.99
S1	126 (39.4)	329.34 ± 19.40
S2	121 (37.8)	340.14 ± 22.94
S3	61 (19.1)	345.62 ± 26.64

Data are presented as mean ± standard deviation (SD). CAP, controlled attenuation parameter; S, histological steatosis grade. CAP values differed significantly across histological steatosis grades (overall $P < 0.001$, one-way ANOVA), with progressively higher mean values observed. Mean CAP values for each histological grade correspond to Supplementary Figure S1.

Supplementary Table S5. Univariable logistic regression analysis of selected candidate predictors for moderate-to-severe steatosis (S ≥ 2).

Variable	OR (Exp(B))	95% CI	*P* value
CAP (dB/m)	1.031	1.018–1.043	< 0.001
ALT	1.019	1.012–1.026	< 0.001
AST	1.033	1.020–1.046	< 0.001
GGT	1.020	1.011–1.029	<0.001
HbA1c	1.429	1.155–1.768	0.001
BMI	1.066	1.021–1.112	0.004
WC	1.025	1.008–1.043	0.004
TC	0.864	0.681–1.097	0.231
HDL-C	0.201	0.064–0.625	0.006
LDL-C	0.922	0.704–1.208	0.557
TG	1.032	0.877–1.215	0.705
Age (years)	0.964	0.935–0.994	0.018

Odds ratios (ORs) with 95% confidence intervals (CIs) are presented. Univariable analyses were used as an initial screening step and were considered together with LASSO results and clinical relevance when constructing the candidate multivariable model. Abbreviations: CAP, controlled attenuation parameter; ALT, alanine aminotransferase; AST, aspartate aminotransferase; GGT, gamma-glutamyl transferase; HbA1c, glycated haemoglobin A1c; BMI, body mass index; WC, waist circumference; TC, tota

l cholesterol; TG, triglycerides; HDL-C, high-density lipoprotein cholesterol; LDL-C, low-density lipoprotein cholesterol.

Supplementary Table S6. LASSO regression-selected candidate predictors for biopsy-confirmed moderate-to-severe hepatic steatosis (S ≥ 2)

**Variable**	**Coefficient (λ_min)**	**Direction**
CAP	0.0258	Positive
ALT	0.0083	Positive
AST	0.0044	Positive
GGT	0.0086	Positive
HbA1c	0.2733	Positive
TC	-0.1619	Negative
TG	-0.1635	Negative
HDL-C	-0.8602	Negative
LDL-C	0 (shrunk to zero)	-
BMI	0 (shrunk to zero)	-
WC	-0.0144	Negative
Age	-0.0103	Negative

LASSO regression was performed with ten-fold cross-validation to select variables associated with moderate-to-severe steatosis (S ≥ 2). Non-zero coefficients at the optimal λ_min were used to identify potentially relevant predictors. Coefficients (λ_min) indicate the shrinkage strength. Direction indicates whether the variable was positively or negatively associated with the outcome. Abbreviations: CAP, controlled attenuation parameter; ALT, alanine aminotransferase; AST, aspartate aminotransferase; GGT, gamma-glutamyl transferase; HbA1c, glycated haemoglobin

A1c; BMI, body mass index; WC, waist circumference; TC, total cholesterol; TG, triglycerides; HDL-C, high-density lipoprotein cholesterol.

Supplementary Table S7. Candidate multivariable logistic regression model for detecting moderate-to-severe steatosis (S ≥ 2).

**Variable**	**β**	**SE**	**OR**	**95% CI**	**P**
CAP	0.0333	0.0082	1.034	1.017–1.051	<0.001
ALT	0.0113	0.0040	1.011	1.004–1.020	0.005
GGT	0.0079	0.0049	1.008	0.998–1.018	0.105
HbA1c	0.2814	0.1142	1.325	1.059–1.662	0.014
Age	-0.0220	0.0178	0.978	0.944–1.013	0.217
WC	-0.0237	0.0126	0.977	0.952–1.001	0.060

Multivariable logistic regression analysis was performed to identify independent predictors of biopsy-confirmed moderate-to-severe hepatic steatosis (S ≥ 2). Candidate variables were selected based on univariable logistic regression, LASSO regression, and clinical relevance. AST was not entered together with ALT in the candidate multivariable model because of its strong correlation with ALT (Spearman r = 0.92; Supplementary Figure S3) and to reduce redundancy. Odds ratios (ORs) with 95% confidence intervals (CIs) and P values are presented. Abbreviations: CAP, controlled attenuation parameter; ALT, alanine aminotransferase; GGT, gamma-glutamyl transferase; HbA1c, glycated hemoglobin A1c; WC, waist circumference; OR, odds ratio; CI, confidence interval.

Supplementary Table S8. Multicollinearity diagnostics for predictors included in the final CAP–ALT–HbA1c model.

Predictor	Tolerance	VIF
CAP	0.964	1.037
HbA1c	0.969	1.032
ALT	0.953	1.049

Tolerance and variance inflation factor (VIF) values were calculated to assess multicollinearity among predictors included in the final model. VIF values < 5 were considered acceptable. Abbreviations: CAP, controlled attenuation parameter; ALT, alanine aminotransferase; HbA1c, glycated haemoglobin A1c; VIF, variance inflation factor.

Supplementary Table S9. Apparent calibration and overall prediction performance of the CAP–ALT–HbA1c model for detecting moderate-to-severe steatosis (S ≥ 2).

Model	Hosmer–Lemeshow P value	Calibration intercept	Calibration slope	Brier score
CAP–ALT–HbA1c model	0.728	0.000	1.000	0.1941

Calibration performance was assessed in the development cohort. The Hosmer–Lemeshow test was used to evaluate goodness-of-fit, and the Brier score was used to quantify overall prediction error, with lower values indicating better predictive accuracy. Calibration intercept and slope represent apparent calibration estimates derived from the development dataset. These estimates reflect apparent calibration in the development cohort. Abbreviations: CAP, controlled attenuation parameter; ALT, alanine aminotransferase; HbA1c, glycated haemoglobin A1c.

Supplementary Table S10. Sensitivity analyses of alternative and adjusted logistic regression models for biopsy-confirmed moderate-to-severe hepatic steatosis (S ≥ 2)

Model	Variables	AUC	95% CI
Final CAP–ALT–HbA1c model	CAP + ALT + HbA1c	0.768	0.717–0.819
Alternative metabolic model	CAP + ALT + HOMA-IR	0.762	0.710–0.814
Dysglycaemia-adjusted model	CAP + ALT + HbA1c + T2DM/IGT status	0.772	0.721–0.823
Sex-adjusted model	CAP + ALT + HbA1c + sex	0.770	0.719–0.821
Fully adjusted sensitivity model	CAP + ALT + HbA1c + T2DM/IGT status + sex	0.772	0.721–0.823

All models were fitted using binary logistic regression, with histologically confir med moderate-to-severe hepatic steatosis (S ≥ 2) as the dependent variable. Predicted probabilities from each model were used for receiver operating characteristic curve an alysis. Dysglycaemia status was defined as the presence of T2DM or IGT. The final C AP–ALT–HbA1c model served as the primary reference model for sensitivity analyse s; the other models were evaluated as alternative or additionally adjusted specification s. Abbreviations: AUC, area under the receiver operating characteristic curve; CI, conf idence interval; CAP, controlled attenuation parameter; ALT, alanine aminotransferase;

HbA1c, glycated haemoglobin A1c; HOMA-IR, homeostasis model assessment of ins

ulin resistance; T2DM, type 2 diabetes mellitus; IGT, impaired glucose tolerance.

Supplementary Table S11. Targeted sensitivity analyses evaluating the incremental contributions of age and AST to the primary CAP–ALT–HbA1c model.

Model	Variables	AUC (95% CI)	Brier score	DeLong *P*	LRT *P*	AIC
Final CAP–ALT–HbA1c model	CAP + ALT + HbA1c	0.768 (0.717–0.819)	0.1941	Reference	Reference	381.21
Age-adjusted model	CAP+ALT+HbA1c+age	0.767 (0.716–0.819)	0.1942	0.921	0.384	382.46
AST-adjusted model	CAP+ALT+HbA1c+AST	0.766 (0.715–0.818)	0.1946	0.492	0.530	382.82

The age- and AST-adjusted models were evaluated as targeted sensitivity analyses because both variables showed signals during initial univariable and LASSO screening. AUCs were compared with that of the primary model using paired DeLong tests. Likelihood ratio tests were used to assess improvement in model fit. Lower Brier scores indicate lower overall prediction error, and lower Akaike information criterion values indicate a more favourable balance between model fit and complexity. AST, aspartate aminotransferase; AUC, area under the receiver operating characteristic curve; LRT, likelihood ratio test; AIC, Akaike information criterion.

Supplementary Table S12. Additional sensitivity analyses evaluating the incremental contribution of GGT to the primary CAP–ALT–HbA1c model.

Panel A. Multivariable associations in GGT-extended models

**Model**	**Predictor**	**β (SE)**	**OR (95% CI)**	***P* value**
Primary + raw GGT	CAP	0.0245 (0.0063)	1.025 (1.012–1.038)	<0.001
	ALT	0.0116 (0.0039)	1.012 (1.004–1.019)	0.003
	HbA1c	0.2366 (0.1083)	1.267 (1.025–1.567)	0.029
	**GGT**	**0.0077 (0.0049)**	**1.008 (0.998–1.017)**	**0.117**
Primary + ln(GGT)	CAP	0.0239 (0.0063)	1.024 (1.012–1.037)	<0.001
	ALT	0.0099 (0.0039)	1.010 (1.002–1.018)	0.010
	HbA1c	0.2164 (0.1081)	1.242 (1.005–1.534)	0.045
	**ln(GGT)**	**0.6391 (0.2710)**	**1.895 (1.114–3.223)**	**0.018**

Panel B. Incremental predictive performance of GGT-extended models

Model	AUC (95% CI)	Brier score	DeLong P	LRT P	AIC
Primary CAP–ALT–HbA1c	0.768 (0.717–0.819)	0.1941	Reference	Reference	381.21
Primary + raw GGT	0.764 (0.713–0.816)	0.1948	0.593	0.102	380.53
Primary + ln(GGT)	0.766 (0.714–0.818)	0.1940	0.847	0.017	377.52

The primary CAP–ALT–HbA1c model served as the reference model. Raw GGT was first added on its original U/L scale to directly evaluate its incremental contribution. Because GGT showed a right-skewed distribution, an additional sensitivity analysis was performed using natural log-transformed GGT [ln(GGT)]. AUCs were compared using paired DeLong tests, and likelihood-ratio tests (LRTs) were used to assess improvement in model fit. Lower Brier scores indicate lower overall prediction error, whereas lower Akaike information criterion (AIC) values indicate a more favorable balance between model fit and model complexity. ORs and 95% CIs in Panel A were estimated using the Wald method. Abbreviations: AUC, area under the receiver operating characteristic curve; AIC, Akaike information criterion; CAP, controlled attenuation parameter; ALT, alanine aminotransferase; HbA1c, glycated hemoglobin A1c; GGT, gamma-glutamyl transferase; OR, odds ratio; CI, confidence interval; LRT, likelihood-ratio test.

Supplementary Table S13. Histological fibrosis distribution and sensitivity analyses of liver stiffness and fibrosis in the CAP–ALT–HbA1c model

Panel A. Histological fibrosis distribution across steatosis grades

Fibrosis stage	n (%)	S0	S1	S2	S3
F0	199 (62.2)	10	83	71	35
F1	97 (30.3)	2	34	40	21
F2	13 (4.1)	0	7	4	2
F3	10 (3.1)	0	2	6	2
F4	1 (0.3)	0	0	0	1
F ≥ 2	24 (7.5)	0	9	10	5
F ≥ 3	11 (3.4)	0	2	6	3

Panel B. Fixed-model extensions with LSM and fibrosis stage

Model	Added predictor OR (95% CI)	P value	AUC (95% CI)	Brier score	DeLong P	LRT P	AIC
Primary CAP–ALT–HbA1c	Reference	—	0.768 (0.717–0.819)	0.1941	Reference	Reference	381.21
Primary + LSM	1.009/kPa (0.959–1.062)	0.717	0.767 (0.716–0.818)	0.1942	0.763	0.716	383.08
Primary + F stage	1.195/stage (0.837–1.707)	0.327	0.769 (0.718–0.820)	0.1935	0.838	0.323	382.24

Panel C. Advanced-fibrosis exclusion sensitivity analyses

Analysis	N	S≥2, n	AUC (95% CI)	Brier score
Full cohort	320	182	0.768 (0.717–0.819)	0.1941
Exclude F ≥ 3	309	173	0.767 (0.714–0.819)	0.1955
Exclude F ≥ 2	296	167	0.763 (0.709–0.817)	0.1963

F stage was obtained from the formal final pathology report. F1 included F1, F1a, F1b, and F1c categories, which were collapsed into a single F1 category for analysis. Advanced fibrosis was defined as F ≥ 3. LSM was evaluated as a fixed extension of the primary CAP–ALT–HbA1c model and was not included in the original LASSO screening. Fibrosis stage was entered as an ordinal variable ranging from F0 to F4, with the odds ratio representing the effect per one-stage increase. AUCs of models fitted to the same 320 participants were compared using paired DeLong tests, and likelihood-ratio tests were used to assess incremental model fit. Because the advanced-fibrosis exclusion analyses involved different sample sizes, their AUCs and Brier scores are presented descriptively and AIC values were not compared across these subsets. Abbreviations: CAP, controlled attenuation parameter; ALT, alanine aminotransferase; HbA1c, glycated hemoglobin A1c; LSM, liver stiffness measurement; AUC, area under the receiver operating characteristic curve; CI, confidence interval; LRT, likelihood-ratio test; AIC, Akaike information criterion.

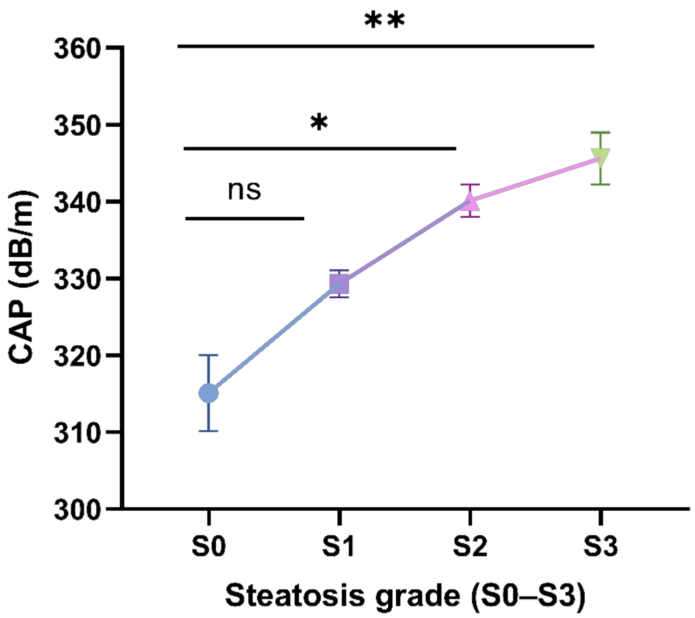

Supplementary Figure S1. Distribution of CAP values across histological steatosis grades (S0 – S3). CAP values increased progressively with histological severity (overall *P* <0.001, one-way ANOVA). Statistical significance between groups was evaluated using post hoc pairwise comparisons: **P* < 0.05, ***P* < 0.01; ns, not significant.

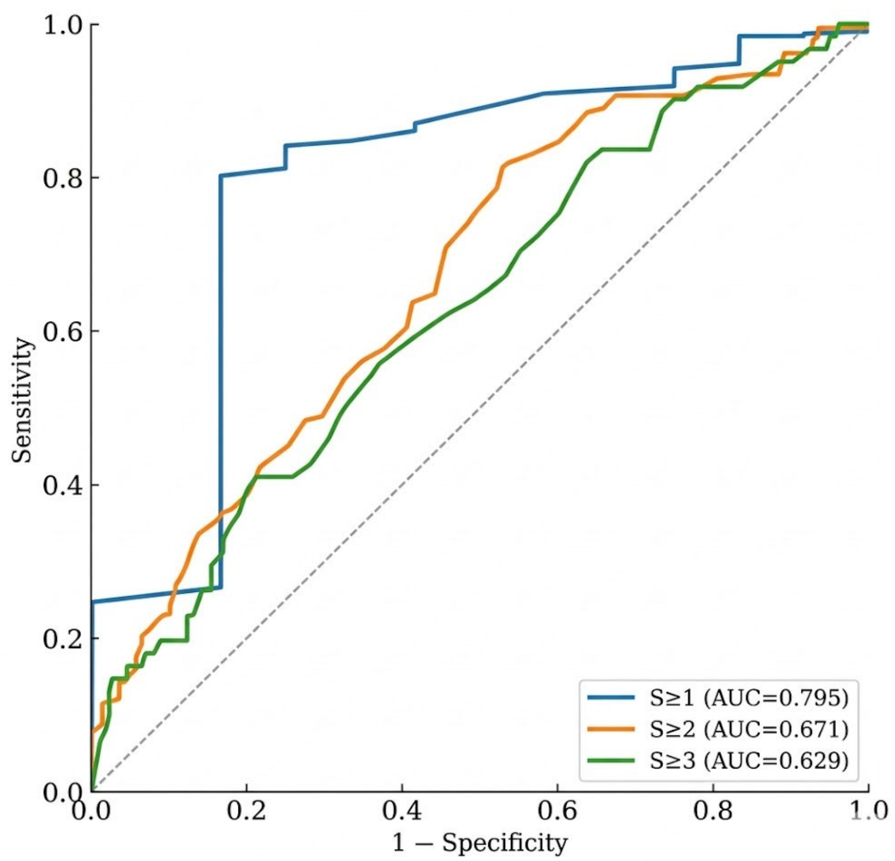

Supplementary Figure S2. ROC curves of CAP for detecting histological steatosis at different thresholds. ROC curves of CAP for detecting histologic steatosis at thresholds S ≥ 1, S ≥ 2, and S ≥ 3, using intraoperative liver biopsy as the reference standard. The area under the curve (AUC) values were 0.795, 0.671, and 0.629 for S ≥ 1, S ≥ 2, and S ≥ 3, respectively, indicating a progressive decline in discriminative performance with increasing steatosis severity.

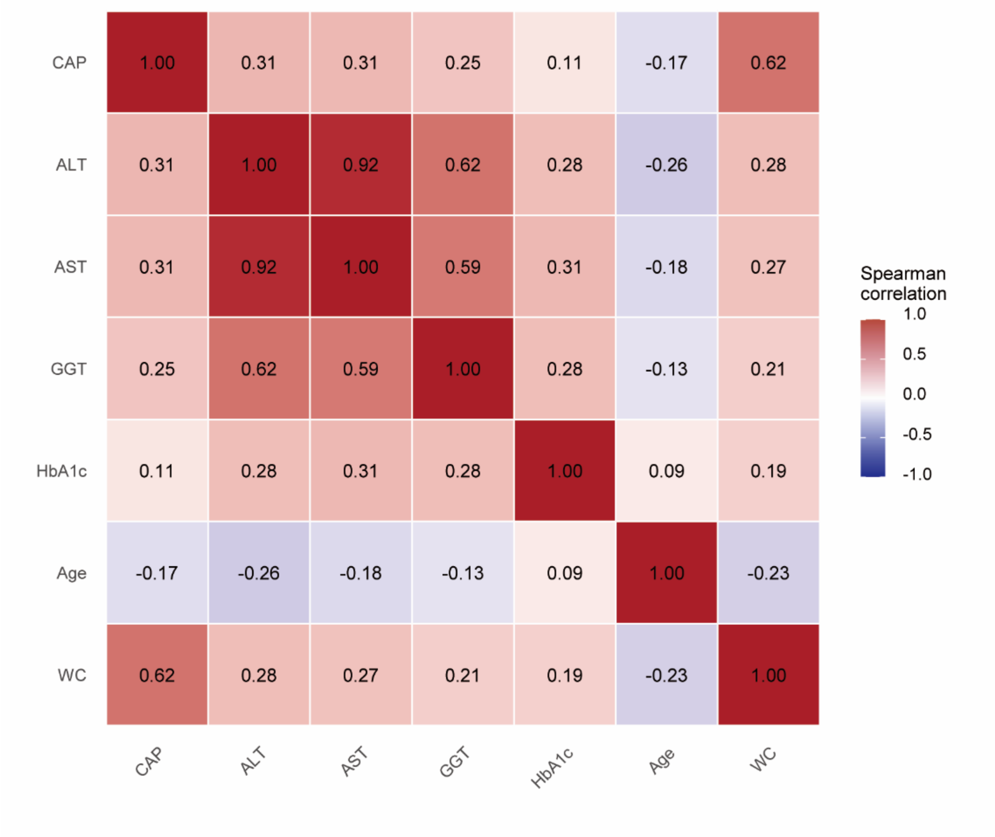

Supplementary Figure S3. Spearman correlation heatmap among candidate predictors for multivariable modelling. The color intensity and numerical values represent Spearman correlation coefficients (r) between candidate predictors. Strong correlation was observed between alanine aminotransferase (ALT) and aspartate aminotransferase (AST) (r = 0.92), whereas other correlations among predictors were moderate. CAP, controlled attenuation parameter; ALT, alanine aminotransferase; AST, aspartate aminotransferase; GGT, gamma-glutamyl transferase; HbA1c, glycated hemoglobin A1c; WC, waist circumference.

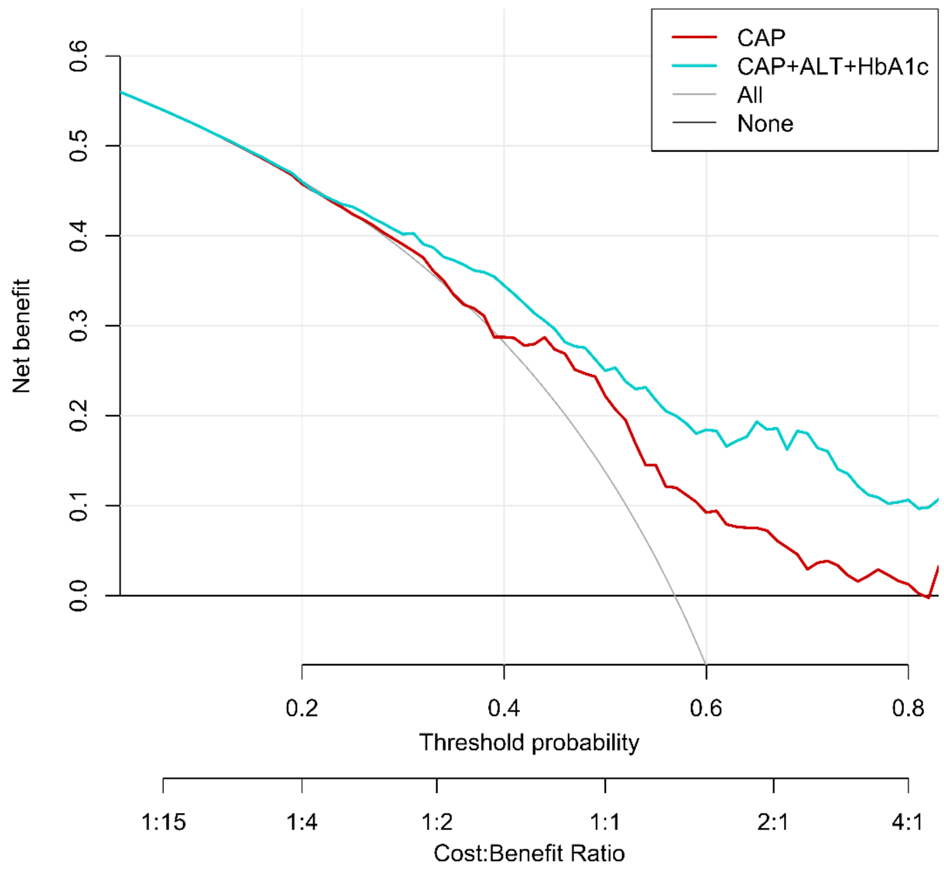

Supplementary Figure S4. Decision curve analysis of CAP alone and the CAP–ALT–HbA1c model. Net benefit was evaluated across threshold probabilities from 0.05 to 0.80. The CAP–ALT–HbA1c model generally showed higher net benefit than CAP alone across threshold probabilities of approximately 0.30–0.80, with greater separation at higher thresholds. The treat-all and treat-none strategies are shown for reference. The clinical action associated with exceeding a threshold was defined as referral for additional liver-focused assessment.

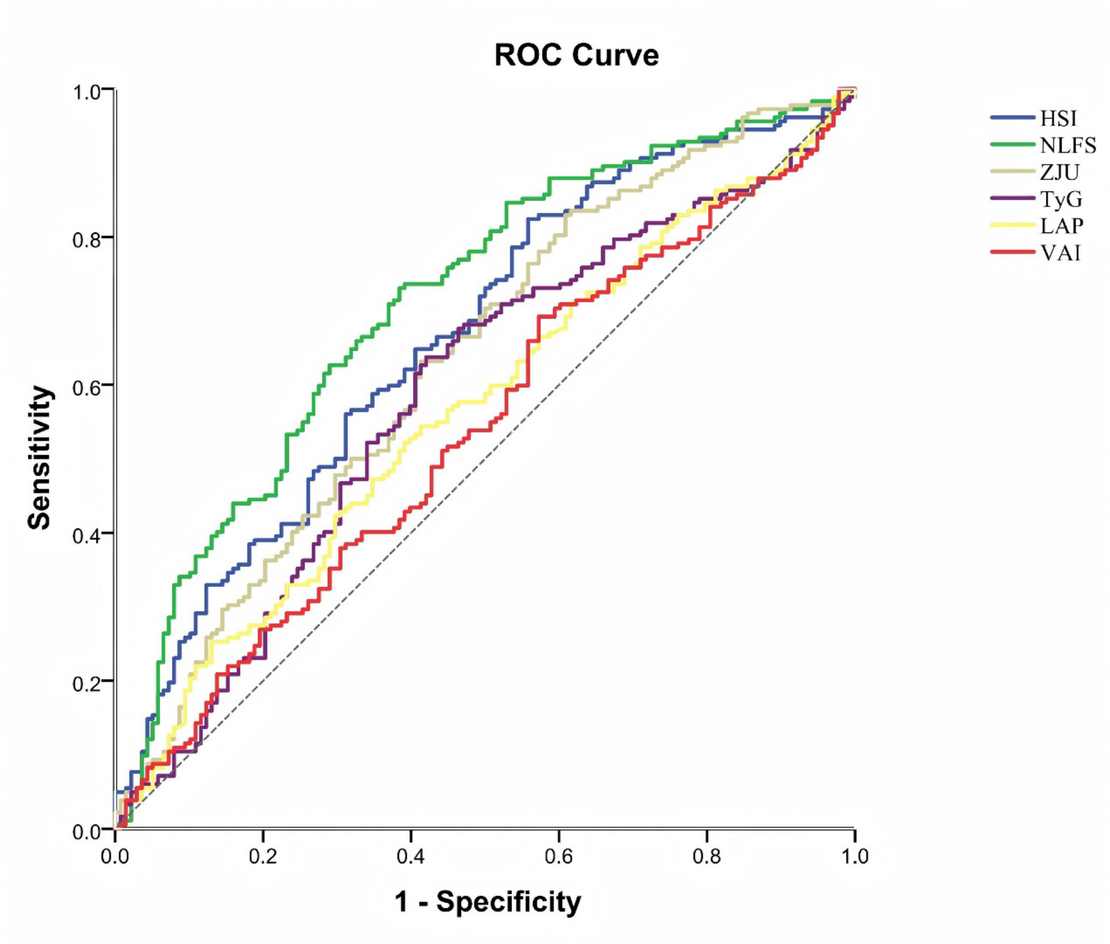

Supplementary Figure S5. ROC curves of conventional metabolic indices for det ecting biopsy-confirmed moderate-to-severe hepatic steatosis. Receiver operating char acteristic curves are shown for the hepatic steatosis index (HSI), NAFLD liver fat scor e (NLFS), ZJU index, triglyceride–glucose index (TyG), lipid accumulation product (LAP), and visceral adiposity index (VAI) for detecting S ≥ 2 steatosis. Among the ev aluated indices, NLFS showed the highest discrimination, followed by HSI. The diago nal dashed line represents discrimination equivalent to chance. HSI, hepatic steatosis i ndex; NLFS, NAFLD liver fat score; ZJU, Zhejiang University index; TyG, triglyceri de–glucose index; LAP, lipid accumulation product; VAI, visceral adiposity index.
